# Integrative analysis of TCGA data identifies miRNAs as drug-specific survival biomarkers

**DOI:** 10.1038/s41598-022-10662-6

**Published:** 2022-04-26

**Authors:** Shuting Lin, Jie Zhou, Yiqiong Xiao, Bridget Neary, Yong Teng, Peng Qiu

**Affiliations:** 1 School of Biological Sciences, Georgia Institute of Technology, Atlanta, USA; 2 Department of Hematology and Medical Oncology, Winship Cancer Institute, Emory University School of Medicine, Atlanta, USA; 3 Department of Biomedical Engineering, Georgia Institute of Technology and Emory University, Atlanta, USA

**Keywords:** Cancer, Computational biology and bioinformatics, Biomarkers

## Abstract

Biomarkers predictive of drug-specific outcomes are important tools for personalized medicine. In this study, we present an integrative analysis to identify miRNAs that are predictive of drug-specific survival outcome in cancer. Using the clinical data from TCGA, we defined subsets of cancer patients who suffered from the same cancer and received the same drug treatment, which we call cancer-drug groups. We then used the miRNA expression data in TCGA to evaluate each miRNA’s ability to predict the survival outcome of patients in each cancer-drug group. As a result, the identified miRNAs are predictive of survival outcomes in a cancer-specific and drug-specific manner. Notably, most of the drug-specific miRNA survival markers and their target genes showed consistency in terms of correlations in their expression and their correlations with survival. Some of the identified miRNAs were supported by published literature in contexts of various cancers. We explored several additional breast cancer datasets that provided miRNA expression and survival data, and showed that our drug-specific miRNA survival markers for breast cancer were able to effectively stratify the prognosis of patients in those additional datasets. Together, this analysis revealed drug-specific miRNA markers for cancer survival, which can be promising tools toward personalized medicine.

## Background

One important aspect of personalized medicine in cancer therapy is the ability to predict an individual patient’s response to drug treatments. Currently, there is an unmet need for such drug-specific predictive biomarkers, which can spare patients from ineffective toxic agents and optimize treatment for individual patients^1^. Utilizing various powerful sequencing technologies, significant efforts have been spent in genomics and proteomics research to profile cancer patients. Such molecular data of cancer enabled studies of the relationship between molecular expression profiles and clinical outcomes, and revealed genetic and epigenetic alterations as biomarkers predictive of survival^2–4^. However, in existing studies, variations in drug treatment among patients were often overlooked, even though it has been reported that drug exposure could affect specific histones, modify gene expression, and impact survival outcomes^5^. In our opinion, there are two main reasons why drug treatment was often not considered in the search for survival biomarkers for cancer. One reason is statistical power. Since larger sample size often leads to improved statistical power for identifying cancer survival biomarkers, studies usually chose to include as many relevant patients as possible, while ignoring the fact that not all included patients received the same drug treatment. The other reason is data availability. In publicly available datasets of cancer genomics and survival studies, drug treatment data of patients is often unavailable. In rare cases where drug treatment data of patients is provided, the data is often in inconsistent and non-standardized formats, making it difficult to use^6^. As a result, the existing biomarkers are often general to the cancer or cancer subtype being studied, but not specific to any drug treatment. Although existing studies have identified useful survival biomarkers, it would be more beneficial if drug-specific survival biomarkers can be identified^7^.

As a valuable data resource for cancer research, The Cancer Genome Atlas (TCGA) provides an opportunity for exploring biomarkers predictive of drug-specific survival outcomes. The TCGA data resources contain comprehensive molecular characterizations of $\sim$11,000 cancer patients across 33 different cancer types, as well as drug treatment data and clinical outcomes of the patients. The molecular characterizations include mutation, copy number variation, methylation, gene expression, miRNA expression and protein expression. Although the drug treatment data in TCGA contains a lot of variations in naming conventions and spelling issues, our group have previously manually standardized the data to eliminate the nomenclature problems^8^. This previous effort enabled integration of the drug treatment data into bioinformatics pipelines for genomics and survival analysis. In this study, we focus on exploring whether miRNAs can be used as drug-specific survival biomarkers, because the potential connection between miRNAs and cancers has been particularly well appreciated by many studies. It has been showed that miRNA involvements in the development of cancer, including cell proliferation, metastasis, differentiation, and evasion of apoptosis^9^. Moreover, it is proposed that expression of more than one third of human genes are under miRNAs control, which means many drug target genes may be regulated by miRNAs^10^. In addition, the functional pattern of miRNA-mRNA interaction network has been shown in the onset and development of cancer^11–13^. Two previous studies took the advantage of network-based models and explored the regulatory mechanism between miRNA and mRNA in colorectal and pancreatic cancer by using the matched specimens of human cancer tissue and adjacent non-tumorous mucosa. Such network-based analysis identified cancer-specific biomarkers, as well as the relationship between deregulated miRNA expression and biological pathways involved in the cancers^14,15^. Since miRNAs play regulatory roles on numerous genes involved in oncology and pharmacology, differences in the levels of circulating miRNAs can contribute to inter-individual variability in response to cancer therapies^16^.

To identify miRNAs that can serve as drug-specific survival biomarkers, we first performed survival analysis on cancer-stratified and drug-stratified subpopulations of patients, and identified miRNAs that significantly correlated with survival outcome in a drug-specific manner. Further, in order to assess the prognostic power of miRNA markers, we investigated the regulatory mechanism of these significant miRNAs by examining their corresponding coding genes. Moreover, we performed a literature survey to assess whether there are existing evidences that supported the correlations between identified miRNAs and drug responses. Finally, we examined several additional breast cancer datasets that provided miRNA expression and survival outcomes, and showed that our drug-specific miRNA survival markers for breast cancer were able to effectively stratify the prognosis of patients in those additional datasets.

## Results

### Identify miRNAs predictive of survival in cancer-drug groups

**Figure 1:**
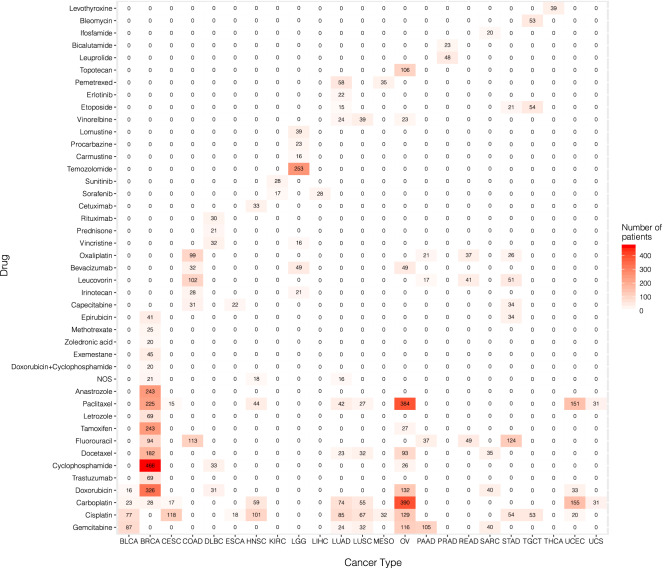
Heatmap of number of patients in each cancer-drug group.

We took patients who suffered from the same cancer type and were exposed to the same drug, and assigned them into one cancer-drug group. A patient can be a member of multiple cancer-drug groups, if the patient received multiple different drugs as reflected in the drug treatment records. The cancer-drug groups containing at least 15 patients with miRNA expression data available were selected for the subsequent analysis to identify miRNA markers for drug-specific survival. A total of 110 cancer-drug groups were selected, which involved 23 cancer types and 43 different drugs. The heatmap in Fig. 1 shows the number of patients in each of the 110 cancer-drug groups. The heatmap was generated by using the R (https://www.r-project.org/, version 4.1.1)^17^ package ’ggplot2’^18^.

For each of the 110 cancer-drug groups, we applied survival analysis for the expression data of each of the 1,881 miRNAs measured in TCGA, which amounted to a total of 206,910 miRNA-cancer-drug combinations examined. For each miRNA, patients in the cancer-drug group were stratified into a highly-expressed class and a lowly-expressed class by applying StepMiner^19^ to the expression data of the miRNA. Specifically, we sorted the expression data across all patients for an miRNA, and then fitted a step function to the sorted data that minimizes the square error between the original and the fitted values, which provides a global threshold to binarize the expression of the miRNA. Since this threshold is derived based on the data of all patients across all cancer types, it is able to robustly define high and low expression of the miRNA. In order to avoid situations where the sample size is too small to extrapolate significant findings, we only performed survival analysis on those miRNAs with at least 5 low-expressed patients and 5 high-expressed patients within the cancer-drug group, which excluded 16,535 out of 206,910 miRNA-cancer-drug combinations in our analysis. Log-rank test was applied to evaluate whether there is significant difference in survival between the two classes. Benjamini-Hochberg multiple tests were used to calibrate the false discovery rate (FDR). MiRNAs with adjusted FDR < 0.1 were selected as predictive markers whose expression levels were predictive of patients’ survival outcome in the corresponding cancer-drug groups. In order to assess whether the identified miRNAs are specifically associated to the drug, we performed the same analysis on all patients in the corresponding cancer type, and only selected the miRNA markers significant in the cancer-drug groups, but not significant in the corresponding cancer type.

In total, we identified 115 significant miRNA-cancer-drug combinations in 44 cancer-drug groups (Table 1) with an FDR threshold of < 0.1, which involved 71 unique miRNAs that were significant in at least one cancer-drug group. All the identified miRNAs significantly associated with survival outcome of specific cancer in a drug-specific manner. We found 10 significant miRNAs in the sarcoma (SARC)-gemcitabine group and 9 significant miRNAs in the ovarian serous cystadenocarcinoma (OV)-paclitaxel group. 31 identified miRNAs were found to be significant in more than one cancer-drug group. These miRNAs are able to predict the survival of patients with different cancers and treated with different drugs, which may be promising biomarkers for drug response in multiple cancer contexts. Among these 31 repeated miRNA markers, two miRNAs were significantly associated with survival in four cancer-drug groups. We observed that the expression level of miR-577 was related to the outcomes of patients with breast cancer (BRCA) and treated with docetaxel, tamoxifen, anastrozole, and NOS, respectively. Another observation was miR-576, which was found to be associated with drug response to fluorouracil, leucovorin in rectum adenocarcinoma (READ), and gemcitabine, docetaxel in SARC.

**Table 1:** Significant miRNAs identified in cancer-drug groups.

Cancer type	Drug	miRNA	Literature	PubMed
BLCA	Carboplatin	1	1	28459431
	Cisplatin	2	4	25843291;26801660;27554045;
				30885939
	Gemcitabine	5	5	32104074;26036346;22132977;
				26606261;30887178
BRCA	Anastrozole	2	0	
	Carboplatin	3	1	25824335
	Cyclophosphamide	4	1	30824586
	Docetaxel	1	0	
	Doxorubicin	1	0	
	Epirubicin	1	0	
	Fluorouracil	3	3	32162886;22955854;24460313
	Methotrexate	1	0	
	NOS	5	0	
	Tamoxifen	1	0	
CESC	Cisplatin	2	9	31647948;28751441;21248297;
				22939244;24462518;25843291;
				26801660;27554045;30885939
COAD	Fluorouracil	1	0	
	Oxaliplatin	1	1	34477047
HNSC	Carboplatin	4	3	31487677;31886905;1411628
	Cetuximab	4	0	
	Paclitaxel	3	5	31352515;27338043;27338042;
				31063487;31496800
LGG	Bevacizumab	1	0	
	Carmustine	1	0	
	Lomustine	1	0	
	Temozolomide	3	0	
LUAD	Docetaxel	2	0	
	Gemcitabine	1	0	
LUSC	Gemcitabine	6	3	31632071;21106054;27129291
MESO	Cisplatin	1	0	
OV	Carboplatin	3	3	27873337;23229111;24314246
	Cyclophosphamide	1	0	
	Doxorubicin	1	0	
	Gemcitabine	4	1	32765654
	Paclitaxel	9	4	25155039;29632436;25973036;
				24060847
	Topotecan	1	0	
PRAD	Leuprolide	1	0	
READ	Fluorouracil	2	0	
	Leucovorin	2	0	
SARC	Docetaxel	5	0	
	Gemcitabine	10	6	31563901;32104074;26036346;
				22132977;26606261;30304549
	Ifosfamide	1	0	
STAD	Leuprolide	7	1	27840964
TGCT	Cisplatin	2	3	29565481;32440152;28096802
	Etoposide	2	0	
UCEC	Carboplatin	2	1	29899543
	Paclitaxel	1	0	

This table represents the number of significant miRNAs identified in all cancer-drug groups. The Literature column represents the number of literature found by PubMed search for each combination.

### Literature survey of identified miRNA markers

**Figure 2:**
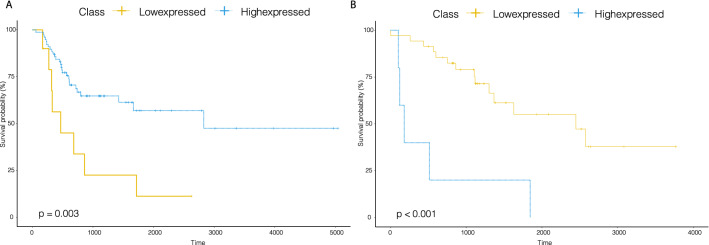
Kaplan–Meier curves of overall survival for patients treated with gemcitabine at low or high expressed classes stratified by miR-330 in the BLCA or SARC.

The significant drug-specific survival miRNA markers included miRNAs that have been previously implicated, as well as the novel ones that have never been mentioned in the literature. The PubMed database was used to search for potentially relevant studies for each of the 115 identified miRNA-cancer-drug combinations, and we have found supportive literature for multiple predictive miRNAs associate with drug responses in various cancer types. The number of papers linked to each identified miRNA-cancer-drug combination is shown in Table 1. For example, two previous studies^20,21^ have reported that the expression of miR-330-5p is related to the promotion of gemcitabine response, one in the context of pancreatic cancer and the other in the context of colon cancer. Coincidentally, we found that increased miR-330 was associated with prolonged survival outcomes of bladder urothelial carcinoma (BLCA) patients exposed to gemcitabine (log-rank, *p*-value= 0.003), and worse survival outcomes of SARC patients exposed to gemcitabine (log-rank, *p*-value< 0.001)(Fig. 2).

**Figure 3:**
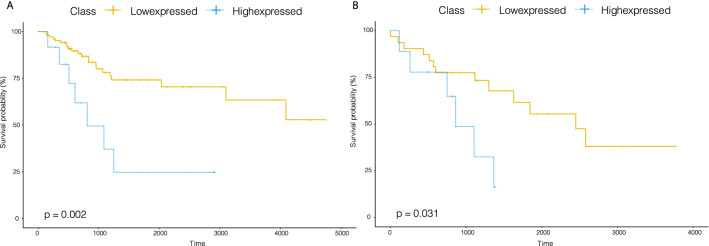
Kaplan–Meier curves of overall survival for patients treated with cisplatin or gemcitabine at low or high expressed classes stratified by miR-296 in the CESE or SARC.

As another example, our analysis suggested that low expression of miR-296 led to increased cisplatin sensitivity in cervical squamous cell carcinoma and endocervical adenocarcinoma(CESE) (log-rank, *p*-value= 0.002) and increased gemcitabine sensitivity in SARC (log-rank, *p*-value= 0.031), as showed in Fig. 3.

**Figure 4:**
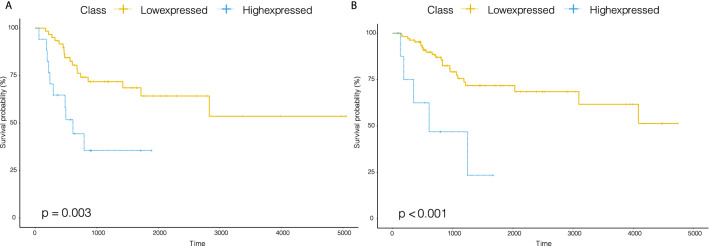
Kaplan–Meier curves of overall survival for patients treated with cis-platin at low or high expressed classes stratified by miR-483 in the BLCA or CESE.

This is supported by several previous studies on the role of miR-296 in drug response^22–25^. One of those studies indicated that miR-296 was able to sensitize lung adenocarcinoma (LUAD) cells to cisplatin in vitro and in vivo^2^. The inhibition of miR-296 also resulted in increased chemosensitivity in esophageal carcinoma (ESCA) cell lines to standard chemotherapeutic agents such as 5-fluorouracil and cisplatin. Another study proved that miRNA-296-5p could promote drug resistance by targeting Bcl2-related ovarian killer, leading to a poor prognosis in pancreatic cancer^26^. As a third example, we found that the over-expression of miR-483 resulted in decreased cisplatin sensitivity in BLCA and CESE (log rank, *p*-value= 0.003; *p*-value< 0.001) (Fig. 4).

This is consistent with previous studies showing that miR-483-5p is significantly associated with cisplatin sensitivity and with overall survival in patients with tongue squamous cell carcinoma (TSCC)^27,28^. In addition, there has been evidence that miR-483-3p expression is increased in platinum drug-resistant ovarian carcinoma cells and indicated an association between increased miR-483-3p expression and cisplatin resistance^29,30^.

### Correlation between drug-specific miRNAs and their target genes

To examine the correlation between the identified drug-specific miRNAs and their associated genes in the cancer-drug groups where the miRNAs were identified, we performed drug-specific survival analysis on the expression data of genes that are targets of the identified miRNAs. For each of the 115 identified miRNA-cancer-drug combinations, we extracted the gene expression data of their target genes for patients in that cancer-drug group. We binarized the gene expression data using the StepMiner, the same method we used to binarize the miRNA expression data. The target genes were determined according to the experimentally verified miRNA-target gene pairs incorporated from multiple databases^31–34^. Similar to the analysis of miRNAs, for each cancer-drug group and each gene under consideration, we stratified patients in the cancer-drug group to highly-expressed and lowly-expressed classes according to the binarized data for the gene, and only performed survival analysis on gene-cancer-drug combinations with at least 5 lowly-expressed patients and 5 highly-expressed patients. A total of 8,513 genes targeted by identified miRNAs were examined in the corresponding cancer-drug groups. Log-rank test was performed to examine whether each of the target genes is significantly correlated with survival outcome in the corresponding cancer-drug groups. We applied FDR adjusted significance *p*-value < 0.01 as thresholds to identify genes whose expression were also predictive of drug-specific survival, in the same context as its associated miRNA. 48 gene-cancer-drug combinations exceeded the FDR threshold, which involved 42 unique genes and 9 cancer-drug groups. Based on the 48 significant gene-cancer-drug combinations, $\sim$37% of the identified miRNA-cancer-drug combinations showed significance in their corresponding genes, leading to 59 miRNA-mediated gene-cancer-drug combinations in total, as shown in Table 2. In order to examine the biological role of the 42 target genes, we performed Gene Ontology (GO)^35^ enrichment analysis and Kyoto Encyclopedia of Genes and Genomes (KEGG)^36^ pathway enrichment analysis by using The Database for Annotation, Visualization and Integrated Discovery (DAVID)^37^, searching against the entire Homo sapiens genome. We used a *p*-value threshold of <0.05 to identify enriched processes and pathways. However, due to the small number of target genes identified, this functional enrichment analysis may not have sufficient statistical power to be conclusive, even though it was encouraging to observe the target genes were involved in relevant biological processes including “developmental growth”, “beta-catenin-TCF complex assembly”, and “negative regulation of transcription from RNA polymerase II promoter”, as well as general pathways such as “MicroRNAs in cancer” and “Pathways in cancer”.

In each of these 59 miRNA-mediated gene-cancer-drug combinations, since both the miRNA and the target gene were predictive of survival difference in the corresponding cancer-drug group, these combinations may reflect the functional role of the miRNA markers in affecting drug response through modulating the expression of their target genes. Interestingly, we observed miR-18a and its target gene SMAD4 were predictive of carboplatin response in HNSC, which was consistent with our previous study on the relationship between SMAD4 and carboplatin response^38^. Additionally, we also identified miR-18a to be predictive of the prognosis of HNSC patients treated with cetuximab. In the literature, it has been showed that SMAD4 down-regulation leads to cetuximab resistance in HNSC cell lines^39^, therefore, we can reasonably hypothesize that the miR18a-SMAD4 axis may be involved in the resistance mechanism of cetuximab response in HNSC.

**Table 2:** Significant miRNA-cancer-drug combinations, and corresponding target genes that are also predictive of survival outcoms.

Cancer type	Drug	Gene symbol	FDR	miRNA	FDR
BRCA	Carboplatin	TRIM4	5.392E−03	miR-96	1.004E−03
	Carboplatin	MED1	5.392E−03	miR-96	1.004E−03
HNSC	Carboplatin	SMAD4	2.811E−03	miR-18a	8.442E−02
	Carboplatin	SON	2.811E−03	miR-18a	8.442E−02
	Carboplatin	TNRC6B	1.458E−03	miR-18a	8.442E−02
	Carboplatin	PRR12	2.954E−04	miR-18a	8.442E−02
	Carboplatin	DPF1	6.489E−03	miR-18a	8.442E−02
	Carboplatin	SENP1	2.376E−03	miR-18a	8.442E−02
	Carboplatin	CACNA2D3	8.704E−03	miR-18a	8.442E−02
	Carboplatin	CIC	4.742E−03	miR-18a	8.442E−02
	Carboplatin	UBXN7	6.489E−03	miR-18a	8.442E−02
	Carboplatin	NFIC	6.145E−03	miR-18a	8.442E−02
	Carboplatin	NIPBL	4.624E−03	miR-18a	8.442E−02
	Carboplatin	EDF1	8.793E−03	miR-18a	8.442E−02
	Carboplatin	HNRNPUL1	1.458E−03	miR-18a	8.442E−02
	Carboplatin	TLE3	1.458E−03	miR-18a	8.442E−02
	Carboplatin	TCP1	8.234E−03	miR-18a	8.442E−02
	Carboplatin	TRAPPC1	6.145E−03	miR-18a	8.442E−02
	Carboplatin	SON	4.128E−03	miR-744	1.568E−02
	Carboplatin	TNRC6B	1.673E−03	miR-744	1.568E−02
	Carboplatin	PSMD4	7.077E−03	miR-744	1.568E−02
	Carboplatin	PRR12	2.712E−04	miR-744	1.568E−02
	Carboplatin	CIC	7.077E−03	miR-744	1.568E−02
	Carboplatin	TLE3	1.673E−03	miR-744	1.568E−02
	Carboplatin	TNRC6B	5.805E−04	miR-1293	3.407E−02
	Paclitaxel	KRAS	4.355E−04	miR-18a	9.724E−02
	Paclitaxel	DICER1	6.334E−03	miR-18a	9.724E−02
	Paclitaxel	TNRC6B	3.988E−04	miR-18a	9.724E−02
	Paclitaxel	MED13L	1.747E−03	miR-18a	9.724E−02
	Paclitaxel	TRAPPC10	2.869E−03	miR-18a	9.724E−02
	Paclitaxel	CACNA2D3	1.295E−03	miR-18a	9.724E−02
	Paclitaxel	NIPBL	5.656E−04	miR-18a	9.724E−02
	Paclitaxel	EDF1	3.024E−04	miR-18a	9.724E−02
	Paclitaxel	TLE3	5.656E−04	miR-18a	9.724E−02
	Paclitaxel	RPL9	4.617E−04	miR-18a	9.724E−02
	Paclitaxel	ZNF770	8.679E−03	miR-18a	9.724E−02
	Paclitaxel	MATR3	6.334E−03	miR-18a	9.724E−02
	Paclitaxel	TRAPPC1	3.024E−04	miR-18a	9.724E−02
	Paclitaxel	HERC1	8.679E−03	miR-18a	9.724E−02
	Paclitaxel	TNRC6B	3.103E−04	miR-362	5.662E−02
	Paclitaxel	MED13L	1.359E−03	miR-362	5.662E−02
	Paclitaxel	HERC1	6.100E−03	miR-362	5.662E−02
	Paclitaxel	TNRC6B	1.724E−04	miR-1293	2.016E−02
	Paclitaxel	KMT2D	9.635E−03	miR-1293	2.016E−02
	Paclitaxel	ATXN2L	8.052E−03	miR-1293	2.016E−02
LGG	Bevacizumab	TMEM205	2.373E−04	miR-935	1.084E−03
	Bevacizumab	CREBBP	4.019E−04	miR-935	1.084E−03
	Carmustine	SHCBP1	8.172E−03	miR-33a	9.814E−02
	Temozolomide	MAFB	1.541E−03	miR-135b	1.196E−03
	Temozolomide	SLC7A1	1.541E−03	miR-135b	1.196E−03
	Temozolomide	CHPF2	1.541E−03	miR-671	6.440E−02
	Temozolomide	CEP85	3.072E−04	miR-671	6.440E−02
	Temozolomide	ZFP36L2	5.837E−04	miR-671	6.440E−02
	Temozolomide	SLC7A1	1.761E−03	miR-671	6.440E−02
	Temozolomide	R3HDM2	1.781E−04	miR-671	6.440E−02
OV	Paclitaxel	PPIL1	7.804E−03	miR-504	4.365E−02
	Paclitaxel	CASP2	4.042E−03	miR-130b	7.904E−02
PRAD	Leuprolide	RRP1B	1.011E−04	miR-331	1.696E−02
SARC	Docetaxel	GNAS	1.666E−04	miR-331	4.551E−02

This table represents the miRNA-mediated gene-cancer-drug combinations in all cancer-drug groups.

### Prognostic performance of drug-specific miRNAs in independent datasets

The prognostic power of the identified drug-specific miRNA biomarkers in survival prediction was further tested in several independent miRNA expression datasets generated by microarray platforms. When searching for independent datasets, we considered cancer-drug groups that contained at least 150 patients and produced at least 1 significant miRNA survival marker. Four cancer types were considered, which were BRCA, brain lower grade glioma(LGG), OV and UCEC. After an extensive search on GEO for datasets that contained both miRNA expression data and survival outcome data of these four cancer types, three appropriate datasets were found (GSE40267, GSE19783, and GSE22220), all of which were on BRCA. Using these datasets, we examined the drug-specific miRNAs we identified in BRCA-Doxorubicin, BRCA-Cyclophosphamide, BRCA-Docetaxel, BRCA-Tamoxifen, and BRCA-Anastrozole groups. In these five BRCA-drug groups, 6 drug-specific miRNAs were identified, which were miR-20b, miR-363, miR-628, miR-7.2, miR-577, and miR-3677 as shown in Table 3. Since expression data for miR-7.2 and miR-3677 were not available in these three datasets, we only examined the remaining 4 drug-specific miRNAs for their ability to predict survival outcomes. For each dataset, we performed survival analysis for each of the 4 drug-specific miRNAs. Patients in the datasets were divided into highly-expressed class and lowly-expressed class by the median of expression value of individual miRNAs, and log-rank test was applied to examine the ability of the drug-specific miRNA markers for stratifying patients with different prognosis.

**Table 3:** Additional GEO datasets that served as independent validation datasets.

Datasets	Platform	Number of patients	Outcome
GSE40267	GPL8227	181	OS
GSE19783	GPL10850	101	DFS
GSE22220	GPL8178	210	DRFS

*OS* Overall survival; *DFS* disease-free survival; *DRFS* distant-relapse free survival.

**Figure 5:**
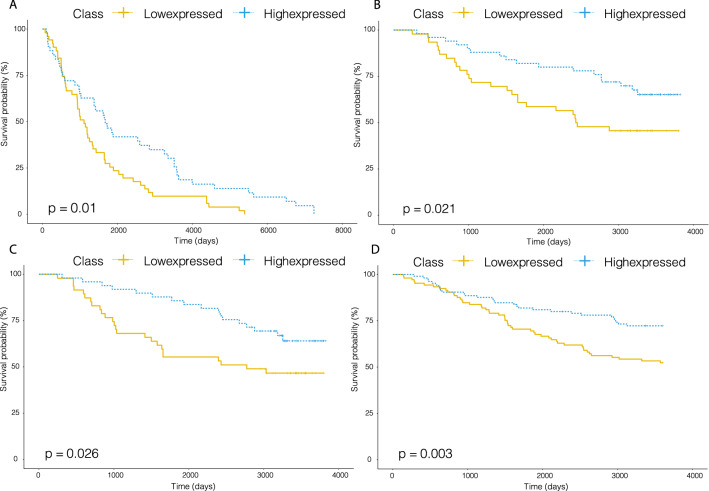
Kaplan–Meyer survival curves of BRCA patients in additional GEO datasets, stratified by drug-specific miRNA marker identified from TCGA data. (**A**) BRCA patients in GSE40267 stratified by miR-363. (**B**) BRCA patients in GSE19783 stratified by miR-20b. (**C**) BRCA patients in GSE19783 stratified by miR-628. (**D**) BRCA patients in GSE22220 stratified by miR-577.

Specifically, in the GSE40267 dataset, stratification of patients by miR-363 expression was significantly correlated with patients’ overall survival (OS) (Fig. 5A, log-rank test, *p*-value = 0.01), where patients with high miR-363 expression had significantly longer overall survival than patients with low expression of miR-363. In the GSE19783 dataset, disease-free survival (DFS) was used as the survival endpoint. High expression of miR-20b and miR-628 were significantly associated with improved survival outcome (Fig. 5B,C, log-rank test, p-value = 0.021 and 0.026, respectively). In the GSE22220 dataset with distant-relapse free survival (DRFS) as the survival endpoint, miR-577 expression was significantly correlated with distant-relapse free survival, where low expression of miR-577 resulted in prolonged survival outcomes of patients (Fig. 5D, log-rank test, p-value = 0.003). Overall, Fig. 5 shows the Kaplan-Meyer curves comparing highly- vs. lowly-expressed patients according to these 4 drug-specific miRNAs. Among the 4 drug-specific miRNAs examined in the three independent datasets, all of them turned out to be significantly predictive of survival outcomes.

## Discussion

This study represents an integrative analysis using TCGA data. By performing survival analysis on miRNA expression data for patients with the same cancer type and exposed to the same drug, we identified several miRNAs significantly associated with survival outcomes in a drug-specific manner. We further investigated the regulatory mechanism of identified miRNAs by examining the relationship between drug-specific miRNA markers and their target genes, which revealed consistencies between the identified miRNAs and their target genes in terms of their expression levels and their correlations with survival outcomes.

For each miRNA-cancer-drug combination being examined, we considered the regulation direction of the miRNA on its target genes, as well as the directions of correlations with the survival outcome. Using the R package ’lm’, we examined the correlation between the expression levels of miRNAs and their target genes in each of the 59 miRNA-mediated gene-cancer-drug combinations in Table 2, and observed that the correlations of all 59 are significant (p-value <0.05). We then examined the consistency of significant miRNAs and their target genes by comparing both their survival directions and regulatory directions. For example, for a given miRNA-mediated gene-cancer-drug combinations where both the miRNA and the target gene were predictive of survival outcome in the same cancer-drug group, we compared the direction of their associations with the survival outcome. If the miRNA inhibits the expression level of the target gene, and high miRNA expression and low gene expression led to better survival, or low miRNA expression and high gene expression led to better survival, then we considered that the miRNA and its target gene were consistent in their directions of correlation with survival outcome. Otherwise, the particular pair of miRNA and target gene showed inconsistency in the directions of correlation with survival outcome. In contrast, if the miRNA activates the target gene, and both the high miRNA expression and high gene expression, or both the low miRNA expression and low gene expression resulted in improved survival outcome, then the miRNA and the target gene were consistent in the directions of survival outcome. Based on the experimentally verified miRNA-target gene pairs we obtained from multiple databases, among the 59 miRNA-mediated gene-cancer-drug combinations, 32 were reported in these databases that the target genes were inhibited by the miRNAs, whereas the regulatory relationships for the rest 27 are unknown. Here, 22 ($\sim$69%) of the 32 with inhibition relationship showed consistency in the survival directions with the miRNAs and target genes, which illustrated the consistency between the drug-specific miRNAs and the target genes regarding their correlations with survival outcome. Although the remaining 10 ($\sim$31%) showed inconsistent survival direction, this may be because gene expression is not only regulated by miRNAs alone, but can be controlled at various steps,including transcription, pre-mRNA splicing and export, mRNA stability, translation, protein modification, and protein half-life^40^.

Some of the identified drug-specific miRNAs (such as miR-296, miR-330 and miR-483) have been previously reported to be potential biomarkers that are highly correlated with drug sensitivity and resistance in various cancer contexts. In several independent datasets on breast cancer, we observed that multiple of our drug-specific miRNA markers for breast cancer (i.e., miR-20b, miR-268, miR-363 and miR-577) were predictive of overall survival and other measures of clinical outcome. Notably, we observed that miR-577 was predictive of survival in multiple BRCA-drug groups (Table 3), indicating that miR-577 may potentially serve as key biomarkers to multiple drug responses in breast cancer. We found that decreased miR-577 expression leads to prolonged survival outcomes of BRCA patients treated with docetaxel, tamoxifen, or anastrozole. However, there is previously literature indicating that down-regulation of miR-577 was correlated with increased invasion and metastasis in breast cancer^41^. Despite this inconsistency in miR-577’s impact on survival between the previous literature and the observations in this study, miR-577 can be a promising biomarker for both predicting clinical outcomes and studying drug mechanisms.

In summary, this study demonstrates that miRNAs can be effective biomarkers predictive of drug-specific survival outcomes in cancer. The miRNA markers we identified are promising and may be useful tools toward personalized medicine.

## Methods

### Data access

MiRNA expression data and gene expression data were downloaded from TCGA Genomic Data Commons (GDC) using the GDC Data Transfer Tool. The miRNA and gene expression data covered $\sim$11,000 patients across 33 cancer types. Clinical data were also downloaded from GDC, which included patients’ drug treatment records and survival outcomes. The drug treatment data contains 10,863 treatment entries for 4,328 patients. The drug names in the treatment records were standardized to remove inconsistency in terms of naming conventions and spelling errors. The standardization was performed based on a manually curated list previously created by our group^8^. The survival data contains the survival outcome for 11,082 patients across 33 cancer types. After removing duplicates in the molecular data and filtering for samples with treatment and survival data, we narrowed down to a total of 9,559 patients across 31 cancer types and 264 unique drugs in this study.

To examine the functional relevance of the identified miRNA markers, we obtained experimentally verified miRNA-target pairs from four databases including mir2Disease, miRecords, TarBase and miRTarBase. The miRNA-target pairs obtained from the four databases include: 96 pairs from mir2Disease, 518 pairs from miRecords, 26388 pairs from TarBase, and 50381 pairs from miRTarBase. These amount to a total of 57,863 human specific miRNA-target gene pairs, involving 14,652 genes and 579 miRNAs.

We also downloaded and log-transformed independent miRNA expression datasets from the Gene Expression Omnibus (GEO, https://www.ncbi.nlm.nih.gov/geo/) database to assess the prognostic power of identified drug-specific miRNAs. A total of 490 patients from 3 independent datasets were analysed in this study. Detailed information about these 3 datasets is shown in Table 4.

**Table 4:** Predictive miRNAs identified in BRCA-drug groups.

Cancer type	Drug	miRNA
BRCA	Doxorubicin	miR-20b
	Cyclophosphamide	miR-20b; miR-363; miR-628; miR-7.2
	Docetaxel	miR-577
	Tamoxifen	miR-577
	Anastrozole	miR-3677; miR-577

### Data preprocessing

The miRNA expression data and gene expression data downloaded from TCGA have been normalized by FPKM-UQ^42^, and we subsequently transformed the expression data by log-transformation. For each miRNA and gene feature, we used StepMiner^19^ to compute a global threshold based on all patients across all cancer types. We first sorted the expression data of a given feature for all patients and then fitted a step function to minimize the square error between the original and the fitted values. Since this threshold is derived based on the data of all patients across all cancer types, it is able to robustly define high and low expression. The threshold of each miRNA or gene was used to binarize the data, so that the patients could be divided into two classes (highly-expressed class vs. lowly-expressed class) according to the binarized data of an individual miRNA or gene feature (Supplementary files 1, 2, 3, 4).

### Survival analysis

We grouped patients based on cancer type and drug exposure. Patients with a given cancer and exposed to a given drug were assigned into one cancer-drug group. For each miRNA, patients in the cancer-drug group were further stratified into highly- or lowly-expressed classes according to the binarized data of that miRNA. Log-rank test was used to assess the statistical significance of the survival difference between highly- and lowly-expressed classes. P-values of the log-rank test were adjusted for multiple testing using Benjamini-Hochberg method with a false-discovery rate (FDR) <0.1. Kaplan–Meier analysis and log-rank test were performed using the R package ‘survival’.

### Literature survey

A literature search was performed using PubMed, accessed via the National Library of Medicine PubMed interface (http://www.ncbi.nlm.nih.gov/pubmed). We programmatically searched the PubMed database with custom Python scripts, using keywords of drugs AND identified drug-specific miRNAs. We searched through PubMed for all keywords in all filed, including the title, abstract and main texts of the articles.

## Supplementary Information

Supplementary Table 1.

Supplementary Table 2.

Supplementary Table 3.

Supplementary Table 4.

## Data Availability

All data used in this analysis can be found at the GDC data portal. The code and processed data were provided as supplementary materials.
